# The Effect of Intra-articular Injection of Autologous Microfragmented Fat Tissue on Proteoglycan Synthesis in Patients with Knee Osteoarthritis

**DOI:** 10.3390/genes8100270

**Published:** 2017-10-13

**Authors:** Damir Hudetz, Igor Borić, Eduard Rod, Željko Jeleč, Andrej Radić, Trpimir Vrdoljak, Andrea Skelin, Gordan Lauc, Irena Trbojević-Akmačić, Mihovil Plečko, Ozren Polašek, Dragan Primorac

**Affiliations:** 1 St. Catherine Specialty Hospital, 49210 Zabok/10000 Zagreb, Croatia; igor.boric@svkatarina.hr (I.B.); eduard.rod@svkatarina.hr (E.R.); zeljko.jelec@svkatarina.hr (Z.J.); andrej.radic@svkatarina.hr (A.R.); trpimir.vrdoljak@svkatarina.hr (T.V.); andrea.skelin@svkatarina.hr (A.S.); 2 Clinical Hospital “Sveti Duh”, 10000 Zagreb, Croatia; 3 School of Medicine, JJ Strossmayer University of Osijek, 31000 Osijek, Croatia; 4 School of Medicine, University of Split, 21000 Split, Croatia; ozren.polasek@mefst.hr; 5 University of Rijeka, Medical School, 51000 Rijeka, Croatia; 6 Genos Glycoscience Research Laboratory, 10000 Zagreb, Croatia; glauc@genos.hr (G.L.); iakmacic@genos.hr (I.T.-A.); 7 Faculty of Pharmacy and Biochemistry, University of Zagreb, 10000 Zagreb, Croatia; 8 School of Medicine, University of Zagreb, 10000 Zagreb, Croatia; mihovil.plecko@gmail.com; 9 Gen-info, 10000 Zagreb, Croatia; 10 Children’s Hospital Srebrnjak, 10000 Zagreb, Croatia; 11 Eberly College of Science, The Pennsylvania State University, University Park, State College, 16802 PA, USA; 12 The Henry C. Lee College of Criminal Justice and Forensic Sciences, University of New Haven, West Haven, 06516 CT, USA

**Keywords:** mesenchymal stem cell, knee osteoarthritis, adipose tissue, regenerative medicine, dGEMRIC, glycosaminoglycans, cartilage

## Abstract

Osteoarthritis (OA) is one of the leading musculoskeletal disorders in the adult population. It is associated with cartilage damage triggered by the deterioration of the extracellular matrix tissue. The present study explores the effect of intra-articular injection of autologous microfragmented adipose tissue to host chondrocytes and cartilage proteoglycans in patients with knee OA. A prospective, non-randomized, interventional, single-center, open-label clinical trial was conducted from January 2016 to April 2017. A total of 17 patients were enrolled in the study, and 32 knees with osteoarthritis were assessed. Surgical intervention (lipoaspiration) followed by tissue processing and intra-articular injection of the final microfragmented adipose tissue product into the affected knee(s) was performed in all patients. Patients were assessed for visual analogue scale (VAS), delayed gadolinium-enhanced magnetic resonance imaging of cartilage (dGEMRIC) and immunoglobulin G (IgG) glycans at the baseline, three, six and 12 months after the treatment. Magnetic resonance sequence in dGEMRIC due to infiltration of the anionic, negatively charged contrast gadopentetate dimeglumine (Gd-DTPA^2−^) into the cartilage indicated that the contents of cartilage glycosaminoglycans significantly increased in specific areas of the treated knee joint. In addition, dGEMRIC consequently reflected subsequent changes in the mechanical axis of the lower extremities. The results of our study indicate that the use of autologous and microfragmented adipose tissue in patients with knee OA (measured by dGEMRIC MRI) increased glycosaminoglycan (GAG) content in hyaline cartilage, which is in line with observed VAS and clinical results.

## 1. Introduction

Articular cartilage is a tissue serving solely mechanical purposes. It enables movement of the joints by absorbing and resisting mechanical loads, while enabling low friction between adjacent joint surfaces at the same time. These unique mechanical properties depend on the organization and composition of the extracellular matrix. Mechanical injury to a joint is the one of the main risk factors for developing osteoarthritis [1]. Bearing in mind that osteoarthritis of the knee has an incidence of 3.4 per 1000 citizens, and a prevalence of 22 per 1000 citizens, according to a recent Dutch epidemiologic study [2], it is not difficult to comprehend the impact that this disease has on the general population [3]. Knee osteoarthritis treatments are a severe burden for society in terms of cost, and there is not much scientific evidence to support available treatment options aside from total knee arthroplasty, in terms of efficacy in preventing further development of the disease. Options such as knee arthroscopy with debridement, corticosteroid injection, and platelet rich plasma (PRP) injection have a limited therapeutic reach [4].

Understanding the biological processes that occur during the deterioration of cartilage in osteoarthritis (OA) is of utmost importance. Articular cartilage contains up to 10% proteoglycan by weight (mainly aggrecan), and its loss is an early sign of osteoarthritis disease. The importance of aggrecan in limb formation in the vertebrae has been studied extensively [5,6]. At the microscopic level, there is evidence of both structural (chondrocyte cell death, duplication of the tidemark, etc.) and biochemical changes (decreased proteoglycan content and altered proteoglycan structure) during the progression of OA [7,8]. Cartilage proteoglycans decrease in size and mass, and their concentration in tissue diminishes with age [9,10]. 

Osteoarthritis is characterized with a low-grade chronic systemic inflammation which has recently acquired a name, “inflammaging” [11,12]. Biomarkers of OA that are present in blood and synovial fluid are of increasing interest, as diagnostics based on clinical examination and radiography have provided little information about metabolic changes [13,14]. As a test for systemic effects of the therapy with injection of micro-fragmented fat tissue on glycosylation we analyzed the composition of the immunoglobulin G (IgG) glycome in both blood plasma and synovial fluid. The IgG glycome composition is known to be a very sensitive biomarker that can be used to evaluate both acute and chronic inflammatory processes [15], and is therefore an excellent tool to follow the systemic effects of therapeutic interventions [16]. 

Several new biological approaches have been developed to overcome the issue of diminished proteoglycan content in osteoarthritic cartilage. Diarthrodial joints like the knee are well suited to intra-articular injection therapy, thus enabling increased bioavailability, reduced systemic exposure, fewer off-target effects, and lower costs. The main disadvantage of all intra-articular approaches is rapid drug clearance via the capillaries in the case of small molecules, and via lymphatic capillaries in the case of macromolecules [17]. Intra-articular half-life for corticosteroids has rarely been found to exceed 12 h [18] and for hyaluronate it is 12–24 h [19]. Intra-articular cell therapies have recently emerged as a method for overcoming unrewarding joint-space kinetics. Mesenchymal stem cells (MSCs) have been under the spotlight in recent years for various regenerative purposes. Their general biological effects are mediated by the secretion of molecules that inhibit ischemia-caused apoptosis and inhibit scar formations, stimulate angiogenesis and vessel stability, and stimulate the mitosis of tissue-intrinsic progenitors [20]. These effects have been recognized in practice for the purpose of knee ligament reconstruction [21] and osteoarthritic cartilage treatment [22,23]. Adipose-derived mesenchymal stem cells (ASCs) have recently attracted attention. In this study, we investigate the effects of an innovative technique developed to obtain microfragmented fat tissue [24]. Bone marrow and adipose tissue are the most readily available sources of MSCs and in this context, adipose tissue is nowadays considered the most promising, due to its abundance, ease of access, and the simplicity of the isolation procedure. In addition, of the many cell types contained in the adipose tissue, MSCs (ASCs) comprise up to 2%, whereas only 0.02% of cells in bone marrow are MSCs. The use of ASCs, either culture-expanded or obtained by mechanical or enzymatic treatment as stromal vascular fraction (SVF), has recently created a huge interest in the context of cartilage regeneration, and shows promising results. However, studies published to date have used a tissue engineering approach, involving the use of scaffolds, cells, and growth factors, either alone or in any combination. In addition to the large number of processing steps, high economic burden and restrictions associated with cell expansion and extensive manipulation, and results achieved to date are far from being completely satisfactory. Therefore, the availability of a minimally manipulated adipose tissue that provides in one step, the key elements to support a natural regenerative response, would have remarkable clinical relevance. Based on these considerations, a commercially available technique that intra-operatively provides microfragmented and minimally manipulated adipose tissue without expansion or enzymatic treatment was used in this study.

We enrolled patients with Kellgren Lawrence osteoarthritis stages III and IV, and followed the effects of single-shot administration of microfragmented fat tissue with adipose-derived stem cells (ASCs) during the course of 12 months. Radiography is currently the gold standard when diagnosing OA in clinical practice. However, radiography is limited by insensitivity to early degenerative changes. In this study, we used delayed gadolinium (Gd)-enhanced magnetic resonance imaging of cartilage (dGEMRIC) in order to assess proteoglycan synthesis. dGEMRIC is a molecular imaging technique that has been used to study glycosaminoglycan (GAG) loss in the articular cartilage of patients with primary OA [25,26], and it can be readily used on patients with cruciate ligament injury [27,28]. With dGEMRIC, T1-maps of hyaline cartilage are created following the intravenous (IV) administration of an anionic gadolinium-based contrast agent gadopentetate dimeglumine (Gd-DTPA^2−^). Since the cartilage matrix is largely composed of GAG molecules with negatively-charged carboxyl and sulfate groups, it repels the negatively charged contrast ions. As a result, the gadolinium concentrations are higher in cartilage regions with low GAG concentrations, and the cartilage T1-relaxation time (T1Gd) is reduced [25]. The Gd-DTPA^2−^ concentration per voxel is described by means of the dGEMRIC index (T1Gd) which is calculated from the five different inversion times using a curve-fitting method. In areas with low GAG, the calculated T1Gd will be low and vice versa. The resulting dGEMRIC index (the average T1Gd in a region of interest) is related to both the GAG concentration and the time between gadolinium administration and image acquisition [29,30,31]. Therefore, healthy cartilage containing an abundance of GAGs will have low concentrations of Gd-DTPA^2−^, whereas degraded cartilage will have high concentrations of the contrast agent in areas where GAGs have been lost. T1-relaxation times are inversely proportional to the concentration of Gd-DTPA^2−^, and thus provide a quantitative metric of cartilage integrity [25,27].

In this study, we hypothesized that there are changes in the composition of the articular cartilage extracellular matrix after administration of autologous and microfragmented fat tissue in patients with knee osteoarthritis that are detectable by means of pre-structural cartilage assessment using magnetic resonance imaging (MRI). To the best of our knowledge, no results have been published on the effects intra-articular administration of autologous and microfragmented fat tissue has on the content of proteoglycans in osteoarthritic cartilage.

Chondrocytes are exposed to side-effects of any drug with an intra-articular administration route. They are sensitive to changes in synovial fluid due to their dependence on diffusion; therefore chondrotoxicity is an important issue. As in any other intra-articular therapy, prolonged efficacy as opposed to fast clearance of the medication is of the highest importance.

We wanted to test whether intra-articular administration of autologous microfragmented fat tissue was not chondrotoxic, and whether it had treatment efficacy during the observed period of 12 months.

## 2. Patients and Methods

### 2.1. Study Design

A prospective, non-randomized, interventional, single-center, open label clinical trial of a single intra-articular injection of autologous microfragmented adipose tissue containing Ad-MSCs in patients with primary knee osteoarthritis was conducted from January 2016 to April 2017 in the St. Catherine Specialty Hospital, Zabok/Zagreb, Croatia. The study protocol was approved by the local Institutional Review Board (IRB) under authorization No: EP 001/2016. The study was registered in ISRCTN (ID: ISRCTN13337022). Patients with primary knee OA who satisfied the inclusion criteria (radiological Kellgren Lawrence grade II-IV; onset of symptoms of the index knee at six or more months ago; ability to follow the instructions of the study; age 40–85) were enrolled in the study. The exclusion criteria were: age < 40 years or > 85 years; chondromatosis or villonodular synovitis of the knee; recent trauma (<3 months) of the symptomatic knee; infectious joint disease; malignancy; pregnancy; patients on anticoagulant therapy with prothrombin time (PT) (<0.70) or suffering from thrombocytopenia and/or coagulation disorder; hypersensitivity to local anesthetics. 

Participants were assessed using a detailed clinical history, complete physical examination and radiological assessment including plain X-rays (AP standing and LL knee projections), full-length weight bearing (FLWB) X-ray in the standing position in order to measure limb alignment, MRI, dGEMRIC and biochemical laboratory test results. Additional assessment included the VAS pain scale. Patients were assessed at time points 0, 3 months, 6 months and 12 months after intervention. A total of 17 patients were enrolled in the study, and 32 knees were assessed. Patients received detailed written and oral information about the study protocol, and were asked to sign an informed consent form. Once they entered the trial, patients were assigned a unique anonymous code, and data were collected in the data logbook. Baseline information collected in the registry included primary diagnoses, medical history, and patient demographics. All procedures were standardized and implemented according to the standard operating procedure protocol (SOP).

### 2.2. Blood and Synovial Specimen Collection and Processing

Blood collections for a serum C-reactive protein (CRP) measurement and plasma blood collection for IgG glycan analyses were taken preoperatively and postoperatively. The blood samples were drawn in two vacutainers, Vacuette^®^ Z Serum Clot Activator Tubes (Greiner Bio-one, Kremsmünster, Austria) for CRP measurements, and Vacuette^®^ EDTA Tubes (Greiner Bio-One) for IgG glycomic analyses. The samples were stored at a temperature of 4 °C before processing in the laboratory. Synovial fluid aspirates for IgG glycosylation analyses were taken during the procedure, stored at (4 °C) and transferred with plasma blood samples to the glyco-laboratory.

### 2.3. Transplantation and Processing of Microfragmented Adipose Tissue with Ad-MSCs

The patients were referred to day surgery unit with an average admission of 3 h. The surgical part of the procedure was set in an operating theatre. Patients were placed in a supine position; the abdominal skin was treated with antiseptic lotion Dermoguard^®^ (Antiseptica, Pulheim, Germany), rinsed with Aqua pro injectione solution (HZTM, Zagreb, Croatia) and dried out and disinfected with Skin-Des^®^ solution (Antiseptica, Pulheim, Germany). The minimal invasive surgical procedure included an infiltration step, in which a total of 250 mL of saline solution prepared with a 40 mL of a 2% lidocaine solution (Lidokain^®^, Belupo, Koprivnica, Croatia) and 1 mL epinephrinhydrochloride (1 mg/mL) (Suprarenin^®^, Sanofi-Aventis, Berlin, Germany) was injected in the abdominal subcutaneous adipose tissue. In the aspiration step, a standard lipoaspiration technique was performed, and the harvested fat was introduced into Lipogems^®^ ortho kit (Lipogems International SpA, Milan, Italy) according to the manufacturer’s instructions as previously described [32]. The collected and processed final microfragmented adipose tissue product was transferred to several 10 mL syringes and injected intra-articularly (4–15 mL) into the index knee. 

### 2.4. Immunoglobulin G Isolation from Plasma and Synovial Fluid Samples

Immunoglobulin G was isolated from blood plasma and synovial fluid samples using protein G agarose beads (20 mg human IgG/mL agarose, Merck, Darmstadt, Germany) using a vacuum manifold (Pall, Ann Arbor, MI, USA). All steps during the isolation procedure were performed at around 50 mmHg. All buffers were filtered through 0.2 μm PES (polyethersulfone) filters (Nalgene Thermo Fischer Scientific, Waltham, ME, USA). Hyaluronidase (HA) from bovine testes (400–1000 units/mg solid, Sigma-Aldrich, St. Louis, MO, USA) was prepared as a stock solution of 1 mg/mL in 1× phosphate buffered saline (PBS), pH 7.4 (137 mmol L^−1^ NaCl, 2.7 mmol L^−1^ Na2HPO4, 9.7 mmol L^−1^ KH2PO4, 2.2 mmol L^−1^ KCl, titrated with NaOH to pH 7.4), and aliquots were stored at −20 °C before use. Synovial fluid samples (100 μL) were pipetted to the previously designated wells of a collection plate, diluted with 690 μL of 1× PBS, buffer (pH 7.4). Then, 10 μL of HA stock solution was added to each synovial fluid sample and incubated at 37 °C for 30 min. After that, samples were shaken at the room temperature for 5 min and incubated at 37 °C for an additional 30 min. Plasma samples (100 μL) were centrifuged for 3 min at 12,100 *g*, pipetted to the previously designated wells of a collection plate and diluted with 1× PBS, pH 7.4 at a ratio of 1:7. All plasma and synovial fluid samples (after treatment with HA) were filtered through 0.45 μm and 0.2 μm AcroPrep GHP (hydrophilic polypropylene) filter plates (Pall) using a vacuum manifold (around 380 mmHg) and immediately applied to equilibrated protein G beads. Protein G agarose beads (65 μL of slurry) were pipetted with a shortened tip to each well of 0.45 μm AcroPrep GHP filter plate (Pall), while protein G agarose was shaken on a shaker. Beads were washed with 4 × 100 μL of 1× PBS (pH 7.4) using a vacuum manifold (<50 mmHg). An AcroPrep GHP filter plate containing the protein G beads was put onto a collection plate; filtered synovial fluid and plasma samples were transferred to the beads and shaken for 5 min on a shaker to facilitate IgG binding to the beads. Unbound proteins were washed away with 3 × 500 μL of 1× PBS (pH 7.4). The AcroPrep GHP filter plate containing Protein G beads was then put on a clean collection plate containing 34 μL of 1 mol L^−1^ ammonium hydrogen carbonate. To elute bound IgG, 2 × 100 μL of 0.1 mol L^−1^ formic acid was added to the beads; the plates were shaken each time for 5 min on a shaker, and IgG was eluted using a vacuum manifold. A volume of 100 μL from each elution fraction was dried in a vacuum centrifuge with a vacuum concentrator (Savant SC210A, Thermo Scientific, Waltham, MA, United States), a refrigerated vapor trap (Savant RVT400, Thermo Scientific, Waltham, MA, United States), and an vacuum pump (OFP400, Thermo Scientific, Waltham, MA, United States), for subsequent *N*-glycan analysis.

### 2.5. *N*-glycan Release, Labeling and Analysis by Ultra-Performance Liquid Chromatography

*N*-glycans from isolated IgG were released with PNGase F (Promega, Madison, WI, USA) and labeled with 2-aminobenzamide (Sigma-Aldrich); excess regents were removed by clean-up using hydrophilic interaction liquid chromatography solid phase extraction (HILIC-SPE), as previously described [33]. Eluates were stored at −20 °C until ultra-performance liquid chromatography (UPLC) analysis. Fluorescently labeled and purified *N*-glycans were separated by HILIC-UPLC using the Acquity UPLC instrument (Waters, Milford, MA, USA) as previously described [33]. *N*-glycan samples were all separated into 24 peaks [34], and the amount of *N*-glycans in each chromatographic peak was expressed as percentage of total integrated area (% area).

### 2.6. Radiography

Plain X-rays (AP standing and LL knee projections) were performed for each patient. Kellgren Lawrence classification was used to assess grade of knee OA using five grades: Grade 0: no radiographic features of OA present; Grade 1: doubtful joint space narrowing (JSN) and possible osteophytic lipping; Grade 2: definite osteophytes and possible JSN on anteroposterior weight-bearing radiograph; Grade 3: multiple osteophytes, definite JSN, sclerosis, possible bone deformity; Grade 4: large osteophytes, marked JSN, severe sclerosis and definitive bone deformity [35]. FLWB X-rays in the standing position were performed in order to measure limb alignment. The alignment was measured as the angle between lines connecting the femoral head and the tibial eminences and the mid of the ankle and the tibial eminences. 

### 2.7. MR Imaging

MR imaging was performed on a 1.5 T magnet (Avanto; Siemens, Erlangen, Germany) using a dedicated knee coil (Siemens, Erlangen, Germany). Five turbo spin-echo inversion recovery sequences (inversion times of 1650 ms, 650 ms, 350 ms, 150 ms and 28 ms; repetition time (TR) = 1800 ms; time to echo (TE) = 19 ms; bandwidth = 326 Hz; field of view (FOV) = 160 mm; matrix = 384 × 384; voxel size = 0.4 × 0.4 × 3 mm; number of excitations (NEX) = 1) were then acquired for subsequent T1Gd mapping to compute the dGEMRIC indices of femoral, tibial and patellar articular cartilage. The severity of early OA in the study cohort was determined according to MRI by an experienced musculoskeletal radiologist using the scoring system introduced by the International Cartilage Research Society (ICRS) based on a modified Outerbridge system divided into five stages according to cartilage lesion size and depth as well as the appearance of the surrounding subchondral bone: Grade 0: normal cartilage; Grade 1: signal intensity alterations with an intact surface of the articular cartilage compared with the surrounding normal cartilage; Grade 2: partial thickness defect of the cartilage with fissures on the surface that do not reach the subchondral bone or exceed 1.5 cm in diameter; Grade 3: fissuring of the cartilage to the level of the subchondral bone in an area with a diameter more than 1.5 cm; Grade 4: exposed subchondral bone [36,37]. The status of the cartilage was analyzed on seven different articular facets: medial and lateral femoral condyle, femoral trochlea, medial and lateral tibial condyle, and both patellar facets. In addition, the thickness of the articular cartilage was measured in the same place before the intra-articular application of stem cells, and in every subsequent MRI examination.

### 2.8. dGEMRIC Protocol

Each subject received gadolinium diethylene triamine penta-acetic acid (Dotarem; Guerbet, Roissy CgG Cedex, Villepinte, France), 0.2 mmol/kg, administered by slow IV infusion through a catheter placed in the antecubital vein with the patient in the supine position in order to avoid thrombophlebitis at the injection site [38]. The relaxivity of the administered MRI contrast agent was the same for all patients because the MRI contrast agent was always applied under the same conditions: contrast agent temperature, magnetic field strength and contrast agent concentration. The contrast agent injection time was less than 5 min. The subject then exercised by walking up and down the stairs for approximately 10 min, starting 5 min after injection, to promote delivery of the contrast agent to the joint. Post-contrast imaging of the cartilage was performed 120 min after contrast administration. The dGEMRIC images were analyzed by an experienced musculoskeletal radiologist using syngoMaplt software (Siemens, Erlangen, Germany). The dGEMRIC index was analyzed on seven different articular facets: the medial and lateral femoral condyle, femoral trochlea, medial and lateral tibial condyle and both patellar facets, before the intra-articular application of stem cells and in any subsequent MRI examination at three, nine, and 12 months after the intra-articular application of stem cells. Regions of interest (ROIs) in which an average T1 index was calculated, were manually drawn to always cover the same central (weight-bearing) part of each articular facet. Articular facets without a cartilage cover where it was not possible to measure the dGEMRIC index were labeled as “0”, and articular facets on which, for some reason, the dGEMRIC index was not measured were labeled as “-“. The highest differences between various measurements within our study at the same ROI were below 6% (range 0.1 to 5.4%), and corresponded with data reported in other studies [38].

### 2.9. Statistical Analysis

We used descriptive and inferential methods to analyze the data. Means and standard deviations were used to estimate central tendency and variability. Numerical data were analyzed using the *t*-test, and the chi-square test was used for categorical variables. We used the pairwise *t*-test for paired and subsequent measurements of the same patient or a knee. Within-individual variation was defined on the basis of the dGEMRIC error rates, which were based on previously published papers, and indicated that the mean difference per region of interest between the two T1Gd measurements ranging from 3.7% to 6.8% [39]. Based on this, we defined the arbitrary change of 15% in subsequent measurements as clinically relevant, and considered this as the liminal value (on the basis of two standard deviations from the estimated error rate). All analyses were performed in R (http://www.r-project.org/), with significance set at *p* < 0.05. 

## 3. Results

### 3.1. Patient Characteristics

Seventeen patients that matched inclusion criteria were consecutively allocated for the study and received intra-articular injection of microfragmented fat tissue with Ad-MSCs. Generally, all the patients enrolled in the study showed similar baseline characteristic of age (mean 69 ± 12), height, weight, body mass index (BMI) and radiographic Kellgren Lawrence grade of osteoarthritis (3 or 4). Sex distribution was: 12 male and five female patients. The pattern, distribution and severity of cartilage tissue deterioration as assessed by MRI and dGEMRIC varied substantially at the baseline. The FLWB X-ray in the standing position revealed mechanical overload patterns of the worn knee compartments with complete lack of the cartilage layer. Out of 32 knees, we observed 25 to have been varus deformities, with an average malalignment of 8.2°; six valgus knees with an average 2° of deformity and one perfectly aligned knee.

### 3.2. Safety and Chondrotoxicity Profile of Intra-Articular Injection of Autologous Microfragmented Adipose Tissue

No adverse events (AE) in the cohort of 17 patients and 32 treated knees with osteoarthritis were observed in association with lipoaspiration or intra-articular (IA) injection. No infectious AEs related to IA injection occurred during follow-up. Exclusion of chondrotoxicity is one of the prerequisites for any successful intra-articular therapy. Instead, we observed an increase in the content of proteoglycans in a percentage of assessed different knee ROI, by means of the dGEMRIC index, which indicates the vitality of chondrocytes and their production of extracellular matrix proteins. This effect is described in detail in the dGEMRIC results section. 

### 3.3. Basic Clinical Outcomes: Visual Analogue Scale for Pain Assessment and C-Reactive Protein

In this study, we measured outcomes of intra-articular injection of autologous and micro-fragmented adipose tissue therapy in 17 patients (32 knees) at baseline (M0), 3 months (M3), 6 months (M6) and 12 months (M12). The initial comparison of the basic clinical parameters indicated a declining pattern of CRP towards mid-time follow-up and a subsequent return to the original values towards the end of follow-up (Table 1). Despite numerically suggestive results, the change was not significant. On the contrary, the pain estimates across the entire study duration showed a significant decrease for both resting and movement estimates (Table 1). 

### 3.4. N-Glycan Profile Analysis

The therapy effect was evaluated on the level of IgG glycosylation by isolating IgG from patient plasma samples at baseline and after 6 and 12 months of follow-up. In addition, IgG was isolated from synovial fluid samples at baseline and after 12 months of follow-up. After releasing and labeling of *N*-glycans, the composition of the IgG glycome isolated from plasma was determined by UPLC analysis, as described in the patients and methods section. The analysis of the *N*-glycan profiles suggested a lack of any significance in paired analyses of the initial vs. the first or second follow-up measurement (Table 2). 

The same situation was observed in the IgG isolated from synovial fluid, where the analysis of the synovial fluid IgG glycome composition at baseline and final measurement (M12) did not yield any significant result (Table 3).

### 3.5. Delayed Gadolinium-Enhanced MRI of Cartilage (dGEMRIC)

In the last step of the analysis, we estimated percentage change of the dGEMRIC results and estimated the number of clinically relevant improvements vs. deteriorations in each patient (Supplementary Figures S1–S15), where a 15% change was considered a relevant change (based on the error rates acquired from the available literature and from our own studies). Out of the 331 total measurements at ROI, we discovered 175 dGEMRIC index values where the GAG results had improved (52.9%), and only 37 (11.2%) deteriorations of relevant GAG with a *p* value of 2.60 × 10^−21^. The results suggest that this method improves GAG content on a significant scale, with over half of the measurements suggesting relevant improvement, and indicates that the method results in only 11% of GAG content decrease, as opposed to the expected GAG decrease over the natural course of the disease. The changes in the cut-off value for relevance did not appear to change the direction or the results magnitude; calculations using cut-off values ranging from 0.07 to 0.30 all suggested significantly more improvements, with the worse *p* value of 4.71 × 10^−9^ (Table 4).

**Figure 1:**
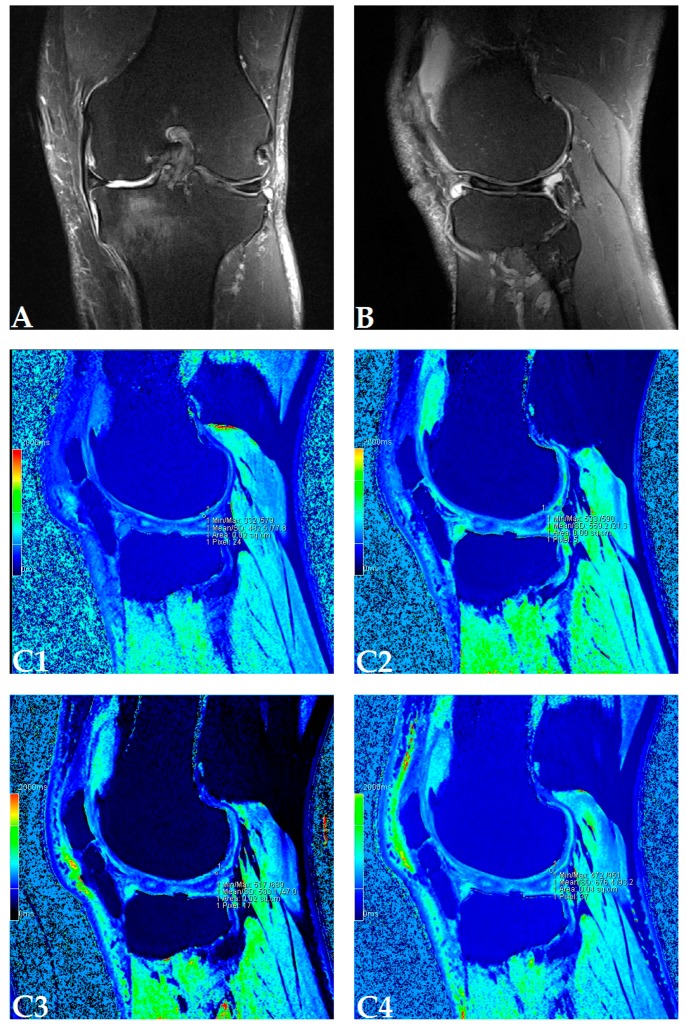

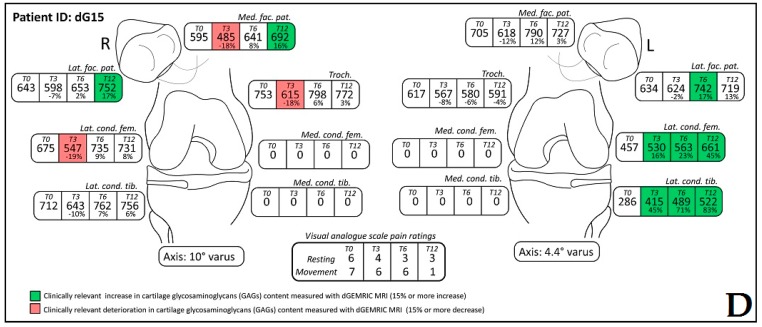
(Patient dG15) Coronal (**A**) and sagittal (**B**) magnetic resonance (MR) images show complete loss of articular cartilage of the medial femoral and tibial condyles (International Cartilage Repair Society (ICRS) grade IV chondromalacia), thinning and shallow fissures of the articular cartilage in the lateral femoral and tibial condyles (ICRS grade IV chondromalacia), edge osteophytes and joint effusion. MR images with the dGEMRIC index values at four-time points (T0: baseline, T3: three months after autologous and microfragmented adipose tissue injection; T6: six months after autologous and microfragmented adipose tissue injection; T12: 12 months after autologous and microfragmented adipose tissue injection) (**C1–C4**). The scheme of the dGEMRIC index with different joint facets throughout the study period at T0, T3, T6 and T12 combined with visual analogue scale (VAS) ratings at T0, T3, T6, T12 (**D**).

**Figure 2:**
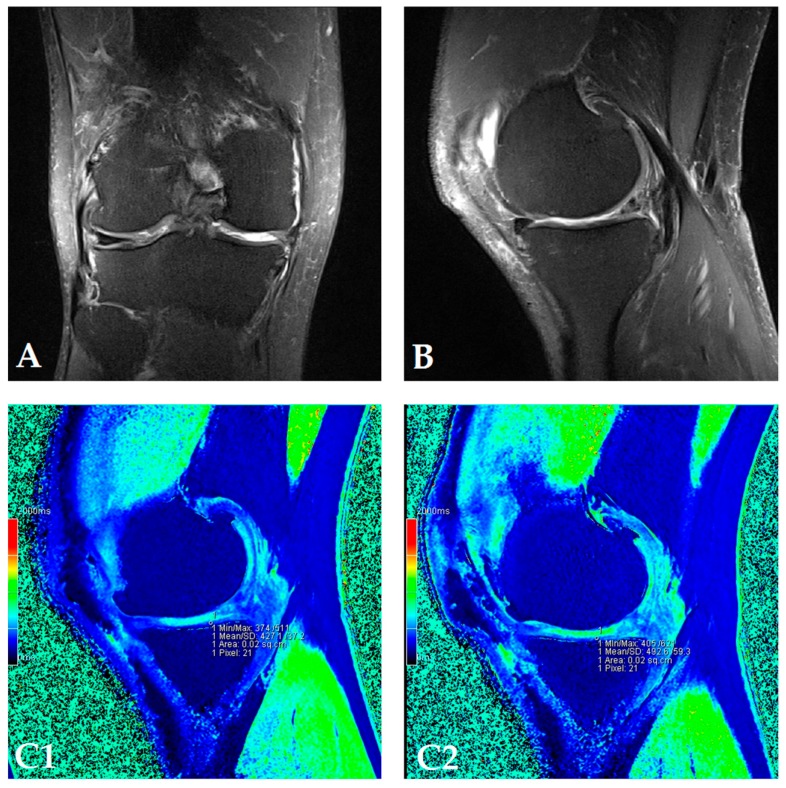

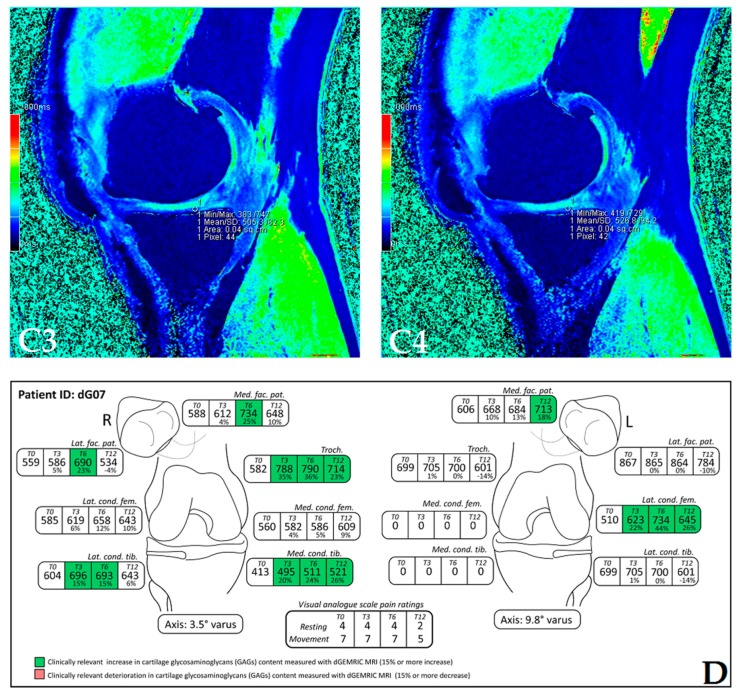
(Patient dG07) Coronal (**A**) and sagittal (**B**) MRI show complete loss of articular cartilage of the medial femoral and tibial condyles (ICRS grade IV chondromalacia), thinning and shallow fissures of the articular cartilage in the lateral femoral and tibial condyles (ICRS grade IV chondromalacia), edge osteophytes and joint effusion. The MRI with the dGEMRIC index values at four-time points (T0: baseline; T3: three months after autologous and microfragmented adipose tissue injection; T6: six months after autologous and microfragmented adipose tissue injection; T12: 12 months after autologous and microfragmented adipose tissue injection) (**C1–C4**). The scheme of the dGEMRIC index with different joint facets throughout the study period at T0, T3, T6 and T12 combined with VAS scale ratings in T0, T3, T6, T12 (**D**).

The dGEMRIC index was measured on seven different articular facets for each knee in the study, and the results are presented in a separate table for each patient (Supplementary Figures S1–S18). The dGEMRIC index was presented in absolute values, and the change of the index value through the study period is shown in percentages in relation to the baseline dGEMRIC index value. However, we observed that there was an increase in index value for the most articular facets after autologous and microfragmented adipose tissue injection. In 120 (53.57%) of 224 joint facets, we observed an increased dGEMRIC index 12 months after autologous and microfragmented adipose tissue injection. As expected, in cartilage lesions International Cartilage Repair Society (ICRS) grade IV, there were no changes in dGEMRIC value through the study period. In 33 (14.73%) joint facets, a decreased dGEMRIC index was found after 12 months in comparison with the baseline. Only in four (1.7%) of those was the decrease greater than 15% (highest in patient dG14, Figure S12). Those were joint facets with ICRS grade III and IV chondromalacia. For example, in patient dG15 (Figure 1D) with 4.4° varus deformity of the left knee, there was a 45% dGEMRIC index increase of the left lateral femoral condyle and an 83% index increase of the left lateral tibial condyle in comparison with the baseline. We also observed an increase of 13% and 3% for the lateral and medial patellar facet, respectively. However, the femoral trochlea index decreased by 4%. In patient dG07 (Figure 2D) with varus knee deformity (left knee 9.8° and right knee 3.5°), we observed a 26% increase of the index value in the medial condyle of the right tibia at the end of the study. Similarly, elevation was observed in other areas. 

## 4. Discussion

Achieving treatment adherence in OA is a great challenge. Our study has shown that intra-articular autologous and micro-fragmented adipose tissue injection in patients with knee osteoarthritis was not associated with any adverse events including chondrotoxicity. These results are in line with other studies demonstrating that the concept of intra-articular administration of ASCs is safe for treating osteoarthritis [22,23]. The novelty of this approach is the use of tissue instead of cells avoiding: (1) regulatory constraints and (2) using a tissue with intact niche. In addition, patients demonstrated significantly improved VAS scores at the 12 month time-point with no significant changes in the CRP parameters. 

The cohort of patients enrolled in the study had knee OA grade III and IV, meaning that we were able to document diverse statuses of the cartilage layer within the joint on MRI scans. We were able to follow the effects of the treatment on full-thickness cartilage layers as well as on completely destroyed cartilage layers with exposed sclerotic subchondral bone. This approach has shown to be very descriptive for evaluating the effects of treatment. The literature explores two potential mechanisms of MSCs for the treatment of OA [40,41]. The first path might be direct differentiation of MSCs in chondrocytes. Though there are studies that have proven that MSCs contribute to the repair of cartilage defects through homing, engraftment, and production of the cartilage extracellular matrix [42,43], we were not able to show that areas of complete cartilage destruction with exposed bone within knee joints were covered with newly-formed cartilage at the end of the 12-month period. Areas of complete cartilage destruction resembled a pattern of mechanical overload in malaligned knees as documented with FLWB X-ray in the standing position. The second therapeutic path of MSCs in OA is paracrine with secretion of bioactive factors. Our study has shown that an efficient response of chondrocytes and proteoglycan synthesis can be expected with the treatment by autologous transplantation of microfragmented fat tissue. Obviously, this tissue serves as a good therapeutic vehicle, leaving ASCs and pericytes viable and effective in their niche [32]. The viability of MSCs and pericytes throughout the surgical procedure is crucial for any expected biological effect. Once the active MSCs and pericytes obtain and sense the surroundings of the targeted knee joint, they start to secrete bioactive factors that are immunomodulatory, antiscarring, antiapoptotic, angiogenic, and trophic (regenerative), meaning that these cells make “therapeutic drugstores” in situ [44]. Major effects of the treatment within our study as measured with dGEMRIC MRI could be observed in the areas of the knee joint where some residual cartilage tissue thickness was present at baseline. 

Articular cartilage in knee OA is subjected to catabolic reactions due to the presence of various cytokines, which may arise from the synovium or from the chondrocytes [45]. These inflammatory cytokines are responsible for the production of proteinases and for downregulation of aggrecan production. Aging causes significant changes in the cartilage extracellular matrix, particularly in the level and structure of proteoglycans. Reduction of cartilage GAGs has been linked with aggrecan and decreases hydration of articular cartilage as well as the ability of the cartilage to respond to mechanical loading [46]. We followed the patients in the study for a period of 12 months, observing a significant increase of the proteoglycan content of the cartilage extracellular matrix. This inevitably reflects the activity of chondrocytes and challenges the natural course of processes related to aging and OA with loss of proteoglycans in the extracellular matrix (ECM). 

Cartilage degeneration due to OA is not visible by radiography until decades after the onset of the disease, when the changes in cartilage are often beyond repair [25,26]. Furthermore, there is only a weak association between radiographic signs of knee OA and its symptoms [27,47]. The Kellgren Lawrence system is the most commonly applied scoring tool used to assess the severity of knee osteoarthritis on a plain radiograph. Soluble drugs in any intra-articular treatment exits joints rapidly [17]. There is evidence that new quantitative MRI techniques can be used to assess the loss of macromolecules such as GAGs in cartilage during the early stages of OA [28]. There are also indications that there is a better correlation between results obtained with these new quantitative MRI techniques and symptoms than between radiographic or routine MRI findings and symptoms [29,30]. In recent years, the dGEMRIC technique has shown potential in evaluating the macromolecular status of normal, degenerated and regenerated articular cartilage. To the best of the authors’ knowledge, this is the first study to evaluate the effect of autologous and micro-fragmented adipose tissue injection on articular cartilage of the knee joint facets using dGEMRIC technique with a 12-month longitudinal assessment of GAG content within the cartilage. The highest differences between various measurements at the same ROI in our study were below 7% (range from 0.1 to 5.4%), which corresponds to published data [38]. There was no influence of the different relaxivity of the administered MRI contrast agent on dGEMRIC index value, because the same MRI contrast agent was always applied, and administration was always performed under the same conditions: contrast agent temperature, magnetic field strength and contrast agent concentration [48]. Each patient had an individual value of dGEMRIC measurement at a specific region of interest in the knee at baseline with respective distribution of load and pattern of cartilage wear. The general trend in OA over the period of 12 months is deterioration of cartilage ECM and decrease [9,46] in proteoglycan content; surprisingly, in our study, only 3.93% of measurements showed a decrease of GAG content with dGEMRIC MRI analysis, given that a 15% change was considered relevant. This result suggests positive effects of ASCs.

A paradigm shift in the diagnostics and follow-up of patients with osteoarthritis is necessary. The golden standard in terms of radiological assessment with standard X-rays of the knee in two planes and Kellgren Lawrence classification is clearly insufficient for the evaluation of effects when treating patients with ASCs.

The IgG glycome is one of the most sensitive biomarkers that changes in numerous different diseases [15]. In general, the composition of the IgG glycome balances chronic inflammation and is believed to contribute to tissue deterioration through the process of inflammaging [49]. Aiming to evaluate effects of the treatment on the systemic level, we have analyzed the composition of the IgG glycome at baseline (before autologous and micro-fragmented adipose tissue injection) and at two follow-up periods. IgG was isolated from the patients’ synovial fluid and blood plasma, and *N*-linked glycans were released and analyzed by UPLC as described in the Patients and Methods section. The comparison of IgG glycome composition before and after autologous and microfragmented adipose tissue injection did not show any statistically significant change that occurred as a result of the treatment. This indicates the absence of any systemic effect on the IgG glycome in either synovial fluid or blood plasma (Table 2 and Table 3). The main significance of this observation is the indication that the observed increase in proteoglycan levels is only local, and a very specific consequence of autologous and micro-fragmented adipose tissue injection without any observable systemic changes in glycosylation. IgG glycome composition is a very sensitive biomarker that changes in multiple conditions, including both chronic and acute inflammation [50,51], this result indicates the absence of inflammatory processes at both local (synovial fluid) and systemic (blood plasma) levels.

In our opinion, the positive effects of autologous and microfragmented adipose tissue injection employed intra-articularly, most likely via trophic and immunomodulatory mechanisms, induce host chondrocytes to proliferate and to produce an extracellular matrix that consequently influences structural and biochemical changes in the cartilage. Moreover, our data suggests that increased GAG production directly influences hyaline cartilage quality, rather than cartilage thickness. The limitation of this study is a lack of comparison with the regular course of cartilage ECM deterioration as measured by dGEMRIC MRI, over a period of 12 months.

Future clinical studies should focus on improving criteria for patient selection. It is not difficult to envision that an observational study following the natural course of knee OA during the 12 months of evaluated by means of dGEMRIC MRI, would be beneficial for strengthening the results of our study.

## Figures and Tables

**Table 1:** Basic clinical comparison across the follow-up.

	Initial (M0)	First Follow-up (M3)	Second Follow-up (M6)	Third Follow-up (M12)	p * (M0–M3)	p * (M0–M6)	p * (M0–M12)
C-reactive protein (CRP); mean ± SD (min-max)	6.54 ± 7.83 (1–20.3)	-	3.86 ± 3.71 (0.6–12)	5.17 ± 5.83 (0.6–23.1)	-	0.158	0.330
Visual analogue scale pain rating, resting; mean ± SD (min-max)	3.94 ± 2.56 (0–8)	1.24 ± 1.48 (0–4)	1.17 ± 1.62 (0–5)	0.56 ± 1.2 (0–4)	0.001	<0.001	<0.001
Visual analogue scale pain rating, movement; mean ± SD (min-max)	7.33 ± 1.72 (4–10)	3.82 ± 2.07 (1–7)	3.67 ± 2.03 (0–7)	3.17 ± 1.98 (0–7)	<0.001	<0.001	<0.001

* *Pair-wise testing with* t*-test for paired samples*; SD: standard deviation; M: months.

**Table 2:** Plasma glycan profiles of the initial measurements, first and second follow-up.

Glycan	Initial Measurement (M0)	First Follow-up (M6)	Second Follow-up (M12)	*p* * (M0–M6)	*p* * (M0–M12)
GP1	0.125 ± 0.063 (0.046–0.277)	0.138 ± 0.055 (0.083–0.258)	0.127 ± 0.052 (0.044–0.254)	0.417	0.996
GP2	1.458 ± 0.97 (0.255–4.007)	1.131 ± 0.632 (0.249–2.167)	1.424 ± 0.955 (0.283–3.999)	0.769	0.523
GP3	0.113 ± 0.02 (0.09–0.151)	0.121 ± 0.021 (0.099–0.161)	0.12 ± 0.023 (0.085–0.162)	0.065	0.142
GP4	27.9 ± 8.246 (12.755–46.784)	29.926 ± 7.142 (22.454–43.436)	28.143 ± 7.034 (14.145–43.06)	0.861	0.957
GP5	0.168 ± 0.028 (0.136–0.218)	0.167 ± 0.027 (0.139–0.21)	0.172 ± 0.032 (0.132–0.237)	0.793	0.366
GP6	7.135 ± 1.299 (4.313–9.109)	7.157 ± 1.683 (4.274–10.042)	7.545 ± 1.663 (4.196–10.429)	0.142	0.050
GP7	0.728 ± 0.679 (0.137–2.773)	0.506 ± 0.207 (0.14–0.759)	0.709 ± 0.634 (0.203–2.717)	0.667	0.575
GP8	18.487 ± 2.753 (13.198–24.336)	17.622 ± 2.368 (13.641–20.534)	18.142 ± 2.626 (13.685–23.972)	0.315	0.074
GP9	9.416 ± 1.873 (7.167–14.423)	9.299 ± 2.26 (7.039–14.446)	9.377 ± 1.8 (6.998–14.379)	0.611	0.562
GP10	4.974 ± 0.983 (4.049–7.72)	5.031 ± 1.159 (3.922–7.585)	5.145 ± 0.991 (4.072–7.557)	0.742	0.374
GP11	0.657 ± 0.082 (0.476–0.756)	0.672 ± 0.093 (0.526–0.785)	0.695 ± 0.13 (0.47–0.977)	0.753	0.169
GP12	0.973 ± 0.773 (0.186–3.166)	0.712 ± 0.298 (0.189–1.078)	0.953 ± 0.732 (0.265–3.121)	0.600	0.152
GP13	0.276 ± 0.049 (0.2–0.356)	0.251 ± 0.041 (0.189–0.31)	0.275 ± 0.046 (0.207–0.37)	0.034	0.841
GP14	10.123 ± 3.266 (3.746–15.708)	9.512 ± 3.073 (4.507–13.431)	9.94 ± 2.932 (4.349–14.829)	0.550	0.691
GP15	1.506 ± 0.27 (0.999–1.974)	1.475 ± 0.253 (1.092–1.867)	1.517 ± 0.221 (1.158–1.875)	0.197	0.786
GP16	2.939 ± 0.592 (2.209–4.089)	2.929 ± 0.613 (2.248–4.007)	2.878 ± 0.547 (2.263–3.912)	0.057	0.143
GP17	0.956 ± 0.186 (0.611–1.159)	0.904 ± 0.181 (0.664–1.186)	0.939 ± 0.194 (0.658–1.232)	0.198	0.992
GP18	6.668 ± 1.498 (3.225–8.798)	6.682 ± 1.786 (3.503–9.145)	6.566 ± 1.449 (3.412–9.026)	0.822	0.961
GP19	1.675 ± 0.534 (0.487–2.582)	1.802 ± 0.366 (1.271–2.326)	1.682 ± 0.482 (0.481–2.416)	0.941	0.755
GP20	0.283 ± 0.066 (0.138–0.393)	0.257 ± 0.055 (0.169–0.33)	0.274 ± 0.06 (0.14–0.343)	0.100	0.460
GP21	0.48 ± 0.106 (0.312–0.577)	0.477 ± 0.114 (0.286–0.647)	0.458 ± 0.091 (0.27–0.578)	0.886	0.151
GP22	0.133 ± 0.049 (0.041–0.191)	0.129 ± 0.037 (0.079–0.19)	0.131 ± 0.04 (0.046–0.178)	0.164	0.765
GP23	1.278 ± 0.414 (0.48–2.001)	1.375 ± 0.432 (0.753–2.048)	1.243 ± 0.437 (0.489–1.996)	0.331	0.903
GP24	1.55 ± 0.542 (0.427–2.266)	1.723 ± 0.39 (1.076–2.238)	1.544 ± 0.521 (0.407–2.515)	0.453	0.748

* Pair-wise testing with *t*-test for paired samples; GP: Glycan peak; M: months.

**Table 3:** Synovial glycan profiles of the initial measurement (M0) vs. final measurement (M12).

Glycan	Initial Measurement (M0)	Final Measurement (M12)	*p* *
GP1	0.16 ± 0.06 (0.09–0.29)	0.16 ± 0.05 (0.09–0.26)	0.698
GP2	1.22 ± 0.61 (0.23–2.06)	1.15 ± 0.51 (0.26–1.91)	0.146
GP3	0.13 ± 0.02 (0.11–0.17)	0.14 ± 0.02 (0.1–0.17)	0.620
GP4	31.05 ± 6.46 (22.45–46.8)	30.45 ± 6.74 (22.52–45.05)	0.945
GP5	0.17 ± 0.03 (0.13–0.26)	0.18 ± 0.03 (0.14–0.27)	0.171
GP6	7.43 ± 1.47 (4.26–10.88)	7.68 ± 1.62 (4.29–10.53)	0.049
GP7	0.52 ± 0.21 (0.12–0.77)	0.51 ± 0.19 (0.15–0.79)	0.514
GP8	17.39 ± 1.98 (13.2–20.59)	17.66 ± 2.2 (13.4–20.54)	0.980
GP9	9.41 ± 1.69 (7.19–14.29)	9.06 ± 1.97 (7.11–14.55)	0.105
GP10	4.89 ± 0.99 (4.08–7.73)	5.3 ± 1.23 (4.02–7.6)	0.158
GP11	0.71 ± 0.11 (0.53–0.99)	0.73 ± 0.12 (0.53–0.98)	0.045
GP12	0.72 ± 0.33 (0.16–1.2)	0.72 ± 0.32 (0.18–1.18)	0.562
GP13	0.28 ± 0.06 (0.18–0.43)	0.28 ± 0.06 (0.18–0.42)	0.541
GP14	8.96 ± 2.58 (3.78–13.55)	9.28 ± 2.76 (4.01–13.61)	0.928
GP15	1.45 ± 0.26 (1.03–2.08)	1.5 ± 0.28 (1.11–1.94)	0.576
GP16	3.01 ± 0.57 (2.27–4.19)	2.85 ± 0.58 (2.25–4.04)	0.152
GP17	0.95 ± 0.17 (0.67–1.21)	0.94 ± 0.18 (0.68–1.31)	0.975
GP18	5.96 ± 1.38 (3.15–8.54)	6 ± 1.52 (3.01–8.26)	0.895
GP19	1.82 ± 0.34 (1.25–2.38)	1.75 ± 0.28 (1.25–2.16)	0.232
GP20	0.31 ± 0.06 (0.22–0.41)	0.33 ± 0.1 (0.2–0.48)	0.664
GP21	0.45 ± 0.07 (0.32–0.55)	0.47 ± 0.18 (0.24–0.94)	0.675
GP22	0.15 ± 0.04 (0.1–0.2)	0.15 ± 0.04 (0.1–0.22)	0.943
GP23	1.17 ± 0.35 (0.57–1.77)	1.07 ± 0.37 (0.53–1.78)	0.015
GP24	1.71 ± 0.4 (1.04–2.33)	1.63 ± 0.36 (1.01–2.35)	0.501

* *t*-test for paired samples.

**Table 4:** Sensitivity analysis for changes of the cut-off values for clinical relevance and corresponding *p* values.

Cut-off	*n* (Improvements)	*n* (Deteriorations)	Chi-Square for Randomness
0.07	175	37	2.60 × 10^−21^
0.15	123	13	4.01 × 10^−21^
0.20	77	8	7.20 × 10^−14^
0.25	57	6	1.32 × 10^−10^
0.30	45	4	4.71 × 10^−9^

## Supplementary Materials

The following are available online at www.mdpi.com/2073-4425/8/10/270/s1, Figure S1: The scheme of the dGEMRIC index with different joint facets throughout the study period at T0, T3, T6 and T12 combined with VAS scale ratings in T0, T3, T6, T12 for patient dG01; Figure S2: The scheme of the dGEMRIC index with different joint facets throughout the study period at T0, T3, T6 and T12 combined with VAS scale ratings in T0, T3, T6, T12 for patient dG02; Figure S3: The scheme of the dGEMRIC index with different joint facets throughout the study period at T0, T3, T6 and T12 combined with VAS scale ratings in T0, T3, T6, T12 for patient dG03; Figure S4: The scheme of the dGEMRIC index with different joint facets throughout the study period at T0, T3, T6 and T12 combined with VAS scale ratings in T0, T3, T6, T12 for patient dG04; Figure S5: The scheme of the dGEMRIC index with different joint facets throughout the study period at T0, T3, T6 and T12 combined with VAS scale ratings in T0, T3, T6, T12 for patient dG05; Figure S6: The scheme of the dGEMRIC index with different joint facets throughout the study period at T0, T3, T6 and T12 combined with VAS scale ratings in T0, T3, T6, T12 for patient dG06; Figure S7: The scheme of the dGEMRIC index with different joint facets throughout the study period at T0, T3, T6 and T12 combined with VAS scale ratings in T0, T3, T6, T12 for patient dG08; Figure S8: The scheme of the dGEMRIC index with different joint facets throughout the study period at T0, T3, T6 and T12 combined with VAS scale ratings in T0, T3, T6, T12 for patient dG09; Figure S9: The scheme of the dGEMRIC index with different joint facets throughout the study period at T0, T3, T6 and T12 combined with VAS scale ratings in T0, T3, T6, T12 for patient dG10; Figure S10: The scheme of the dGEMRIC index with different joint facets throughout the study period at T0, T3, T6 and T12 combined with VAS scale ratings in T0, T3, T6, T12 for patient dG11; Figure S11: The scheme of the dGEMRIC index with different joint facets throughout the study period at T0, T3, T6 and T12 combined with VAS scale ratings in T0, T3, T6, T12 for patient dG12; Figure S12: The scheme of the dGEMRIC index with different joint facets throughout the study period at T0, T3, T6 and T12 combined with VAS scale ratings in T0, T3, T6, T12 for patient dG14; Figure S13: The scheme of the dGEMRIC index with different joint facets throughout the study period at T0, T3, T6 and T12 combined with VAS scale ratings in T0, T3, T6, T12 for patient dG16; Figure S14: The scheme of the dGEMRIC index with different joint facets throughout the study period at T0, T3, T6 and T12 combined with VAS scale ratings in T0, T3, T6, T12 for patient dG17; Figure S15: The scheme of the dGEMRIC index with different joint facets throughout the study period at T0, T3, T6 and T12 combined with VAS scale ratings in T0, T3, T6, T12 for patient dG18.
