# Supplementary figures and images for: The Effect of Intra-articular Injection of Autologous Microfragmented Fat Tissue on Proteoglycan Synthesis in Patients with Knee Osteoarthritis
Source: Genes (Basel). 2017 Oct 13;8(10):270. doi: 10.3390/genes8100270 (PMC5664120; doi:10.3390/genes8100270)

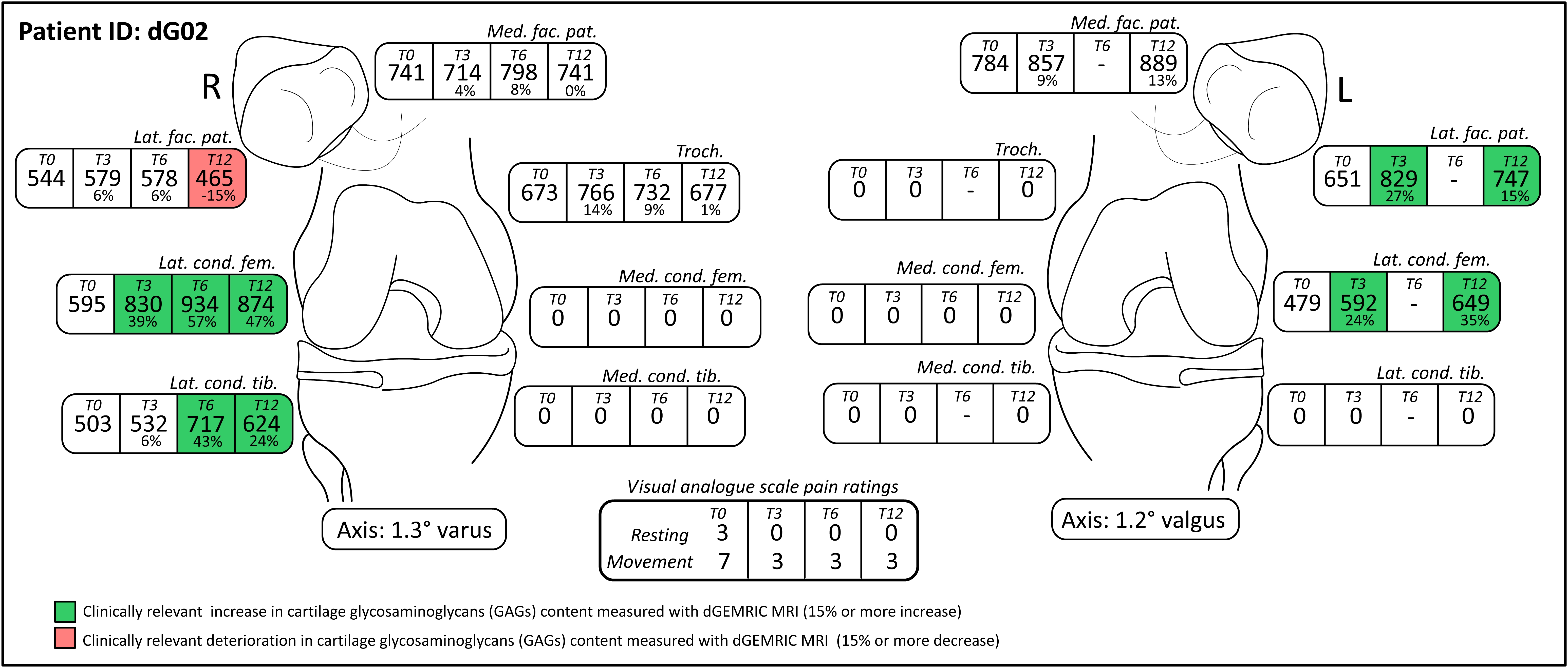

Supplement: Supplementary file 1 [file genes-08-00270-s001.zip › Figure S2.jpg]

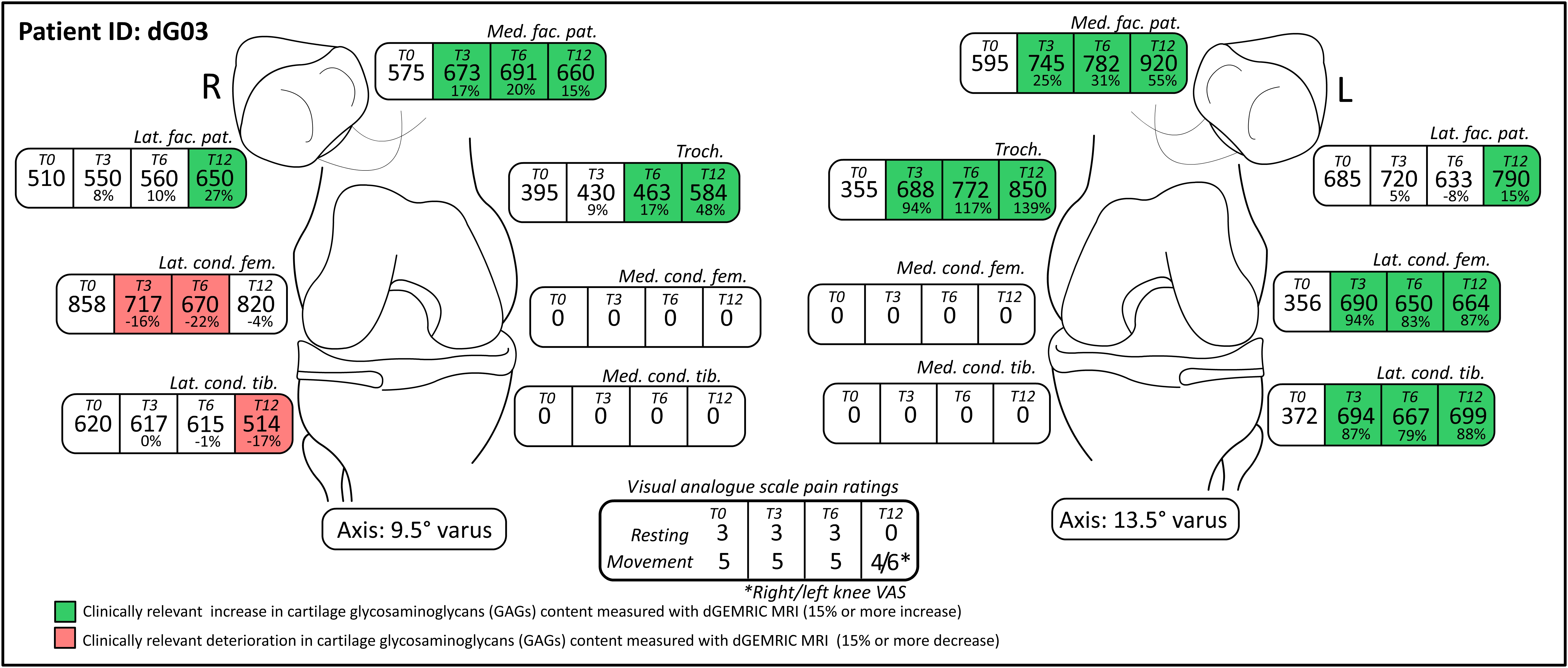

Supplement: Supplementary file 1 [file genes-08-00270-s001.zip › Figure S3.jpg]

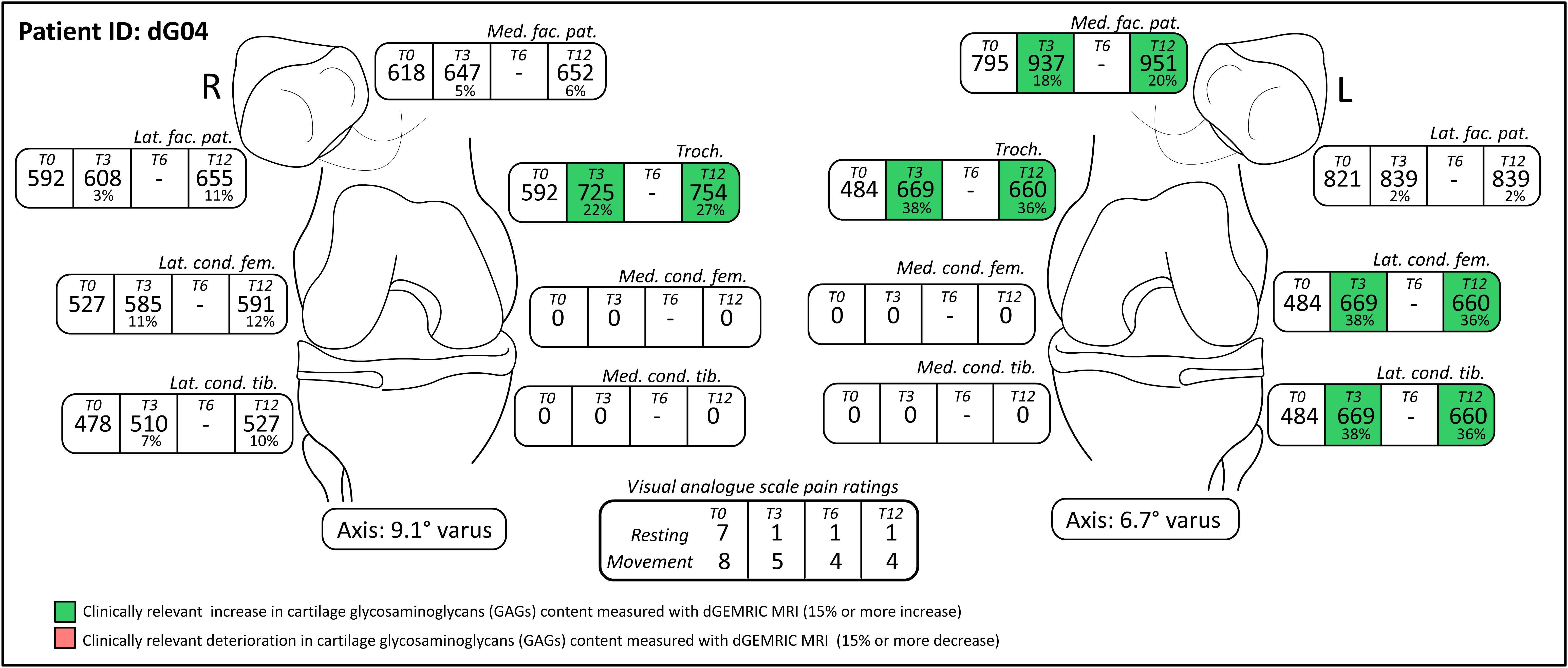

Supplement: Supplementary file 1 [file genes-08-00270-s001.zip › Figure S4.jpg]

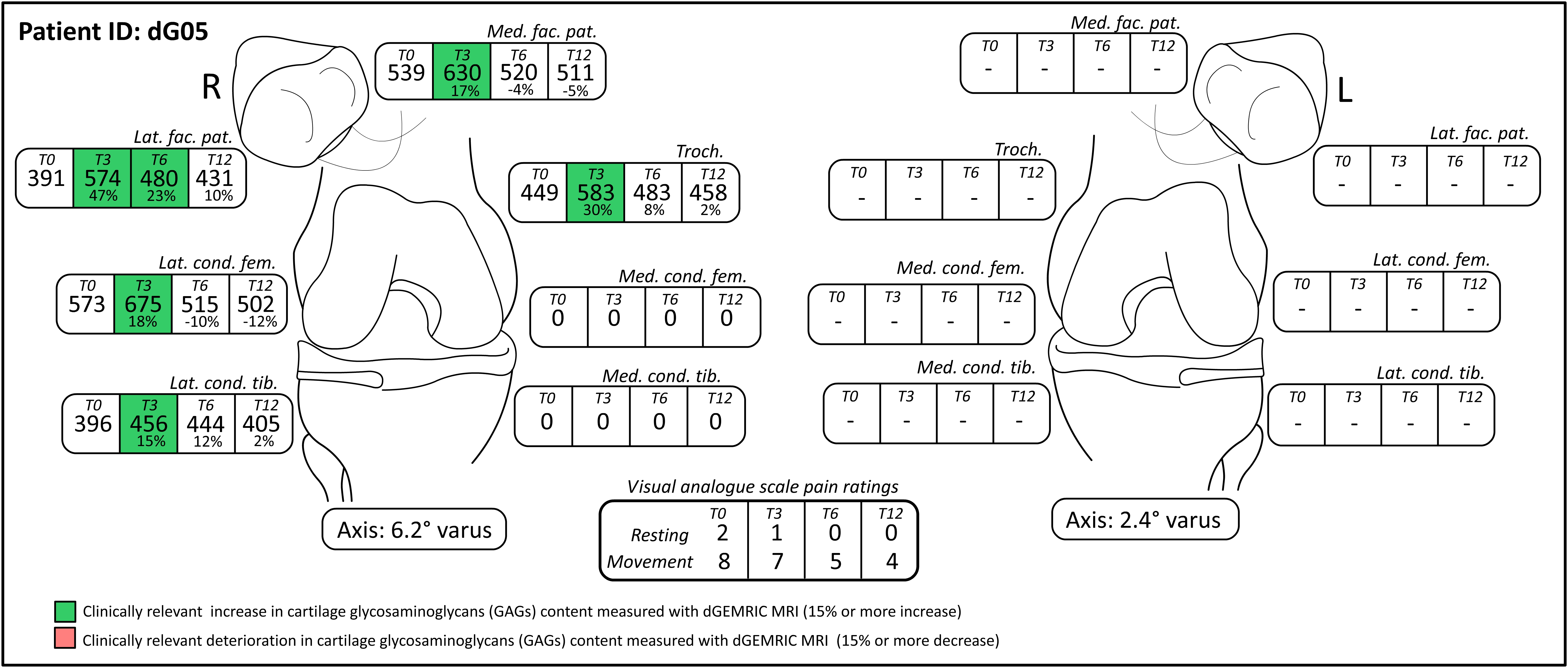

Supplement: Supplementary file 1 [file genes-08-00270-s001.zip › Figure S5.jpg]

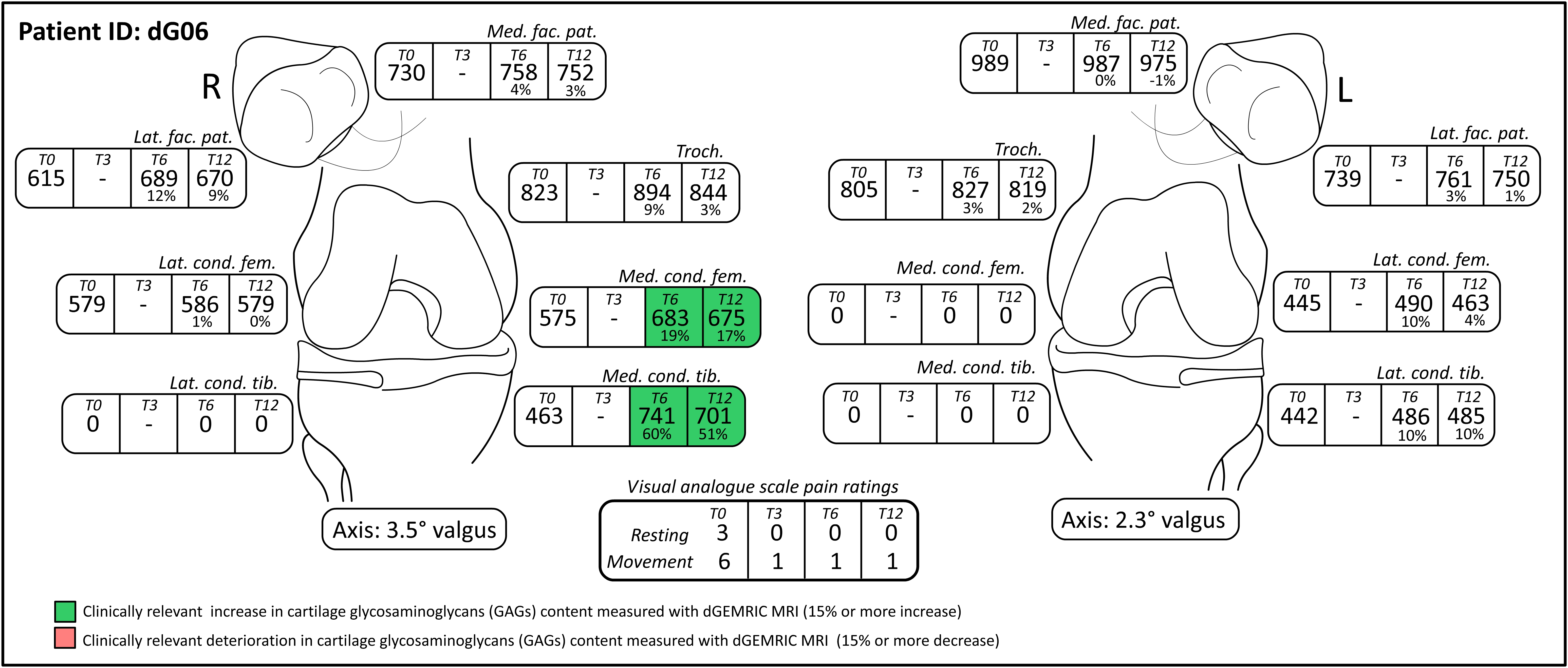

Supplement: Supplementary file 1 [file genes-08-00270-s001.zip › Figure S6.jpg]

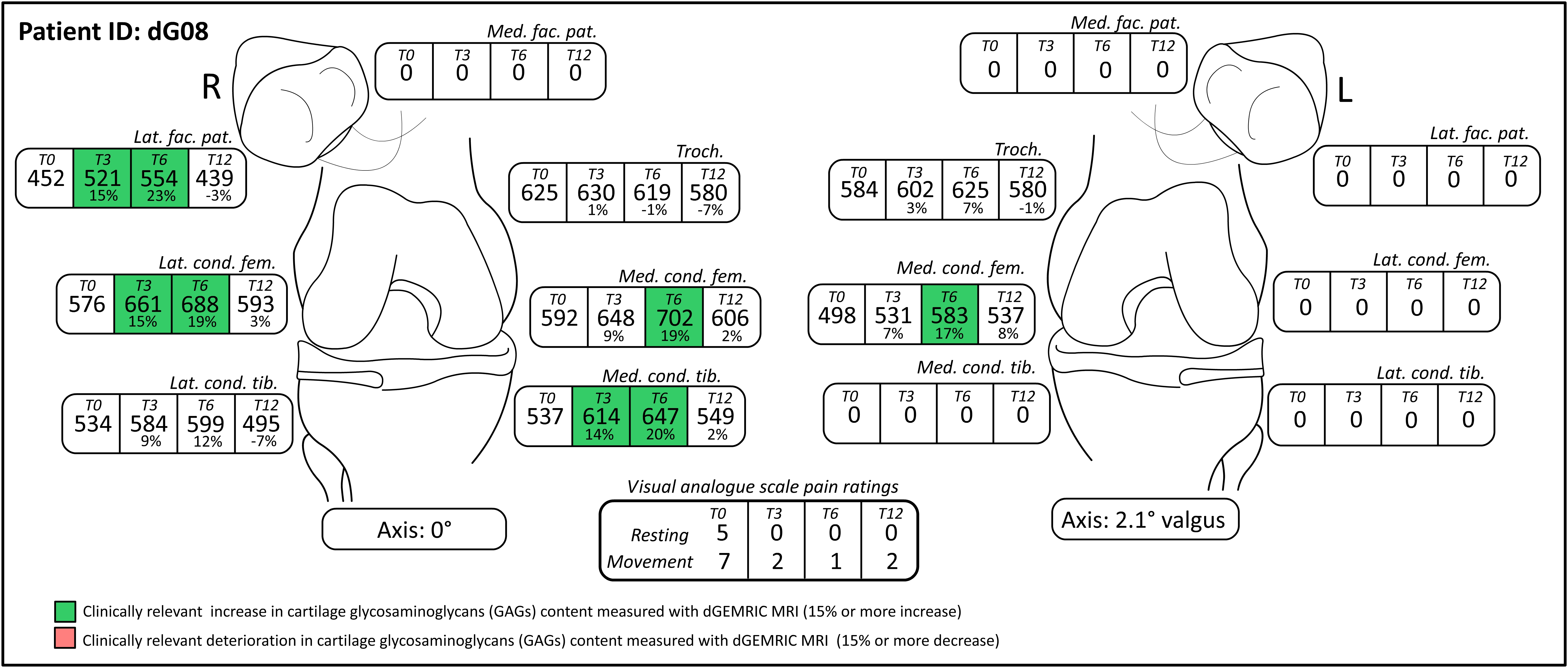

Supplement: Supplementary file 1 [file genes-08-00270-s001.zip › Figure S7.jpg]

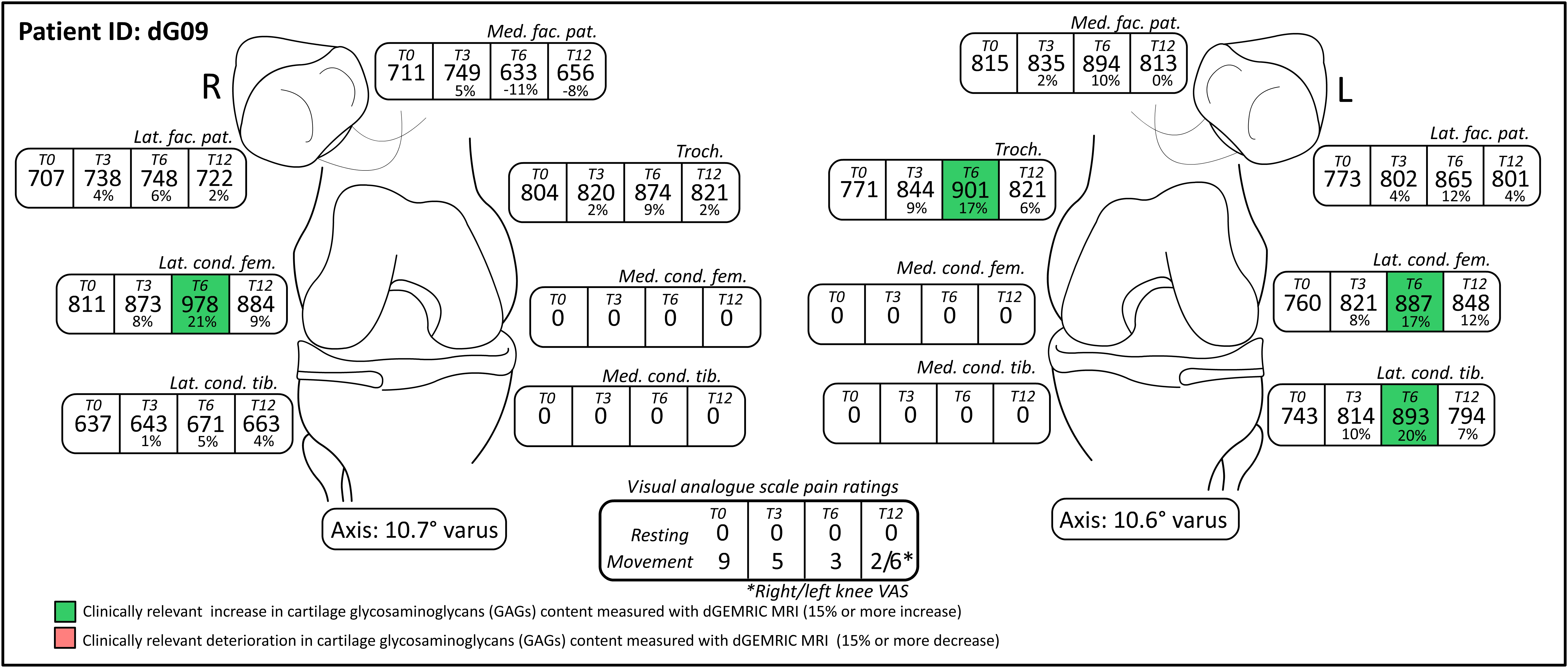

Supplement: Supplementary file 1 [file genes-08-00270-s001.zip › Figure S8.jpg]

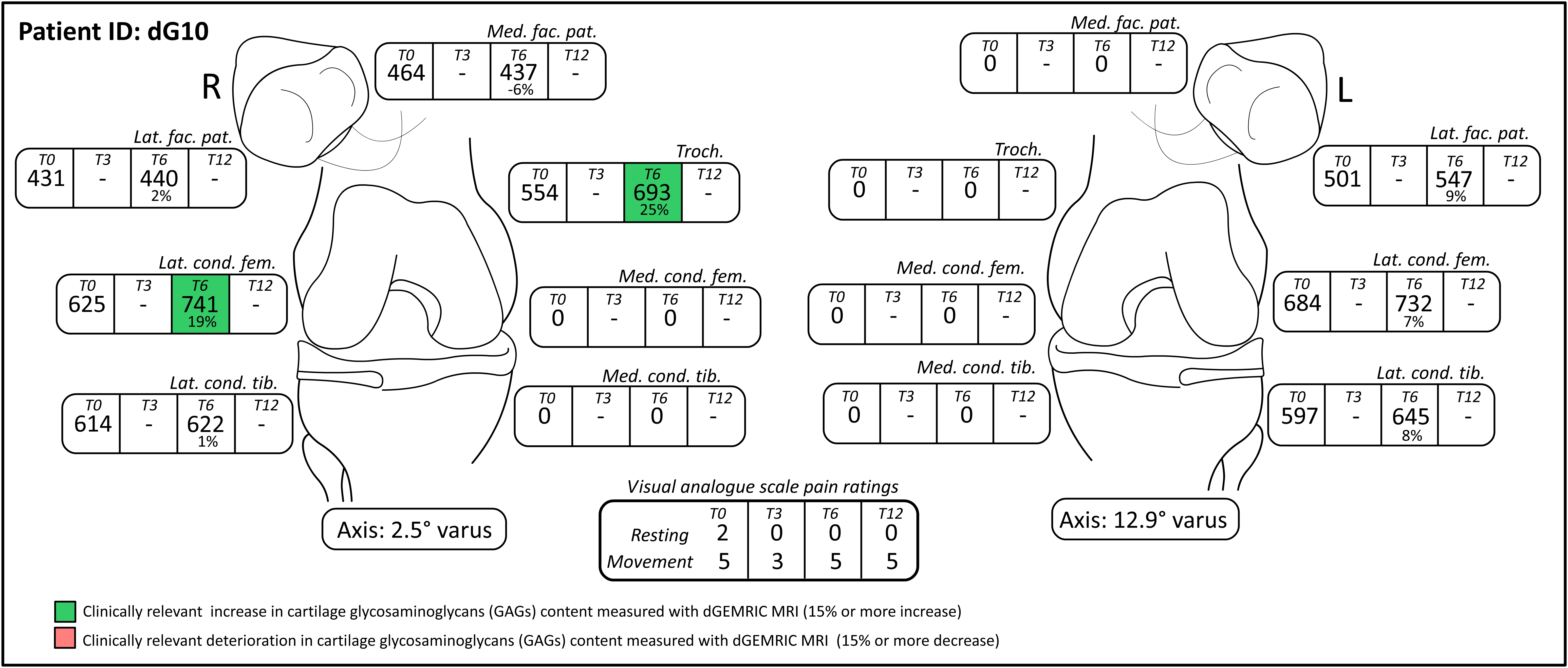

Supplement: Supplementary file 1 [file genes-08-00270-s001.zip › Figure S9.jpg]

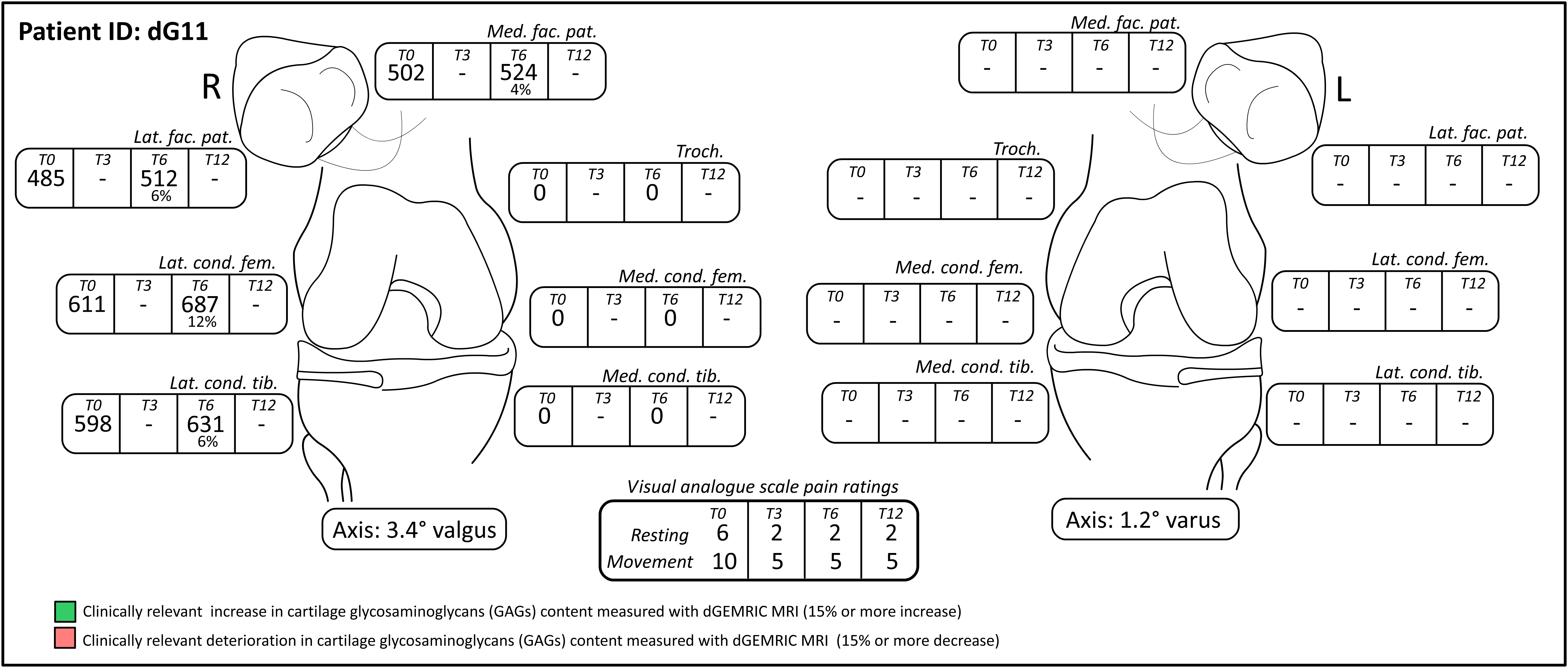

Supplement: Supplementary file 1 [file genes-08-00270-s001.zip › Figure S10.jpg]

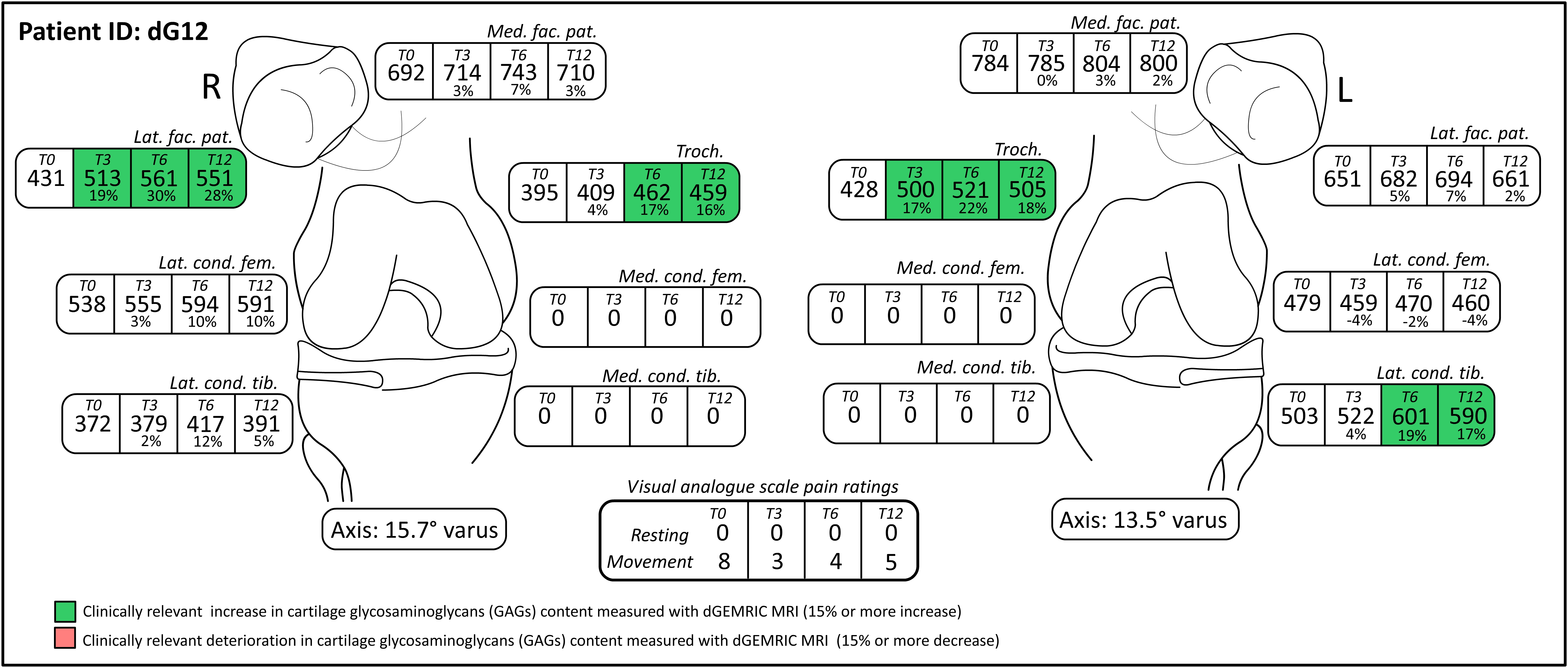

Supplement: Supplementary file 1 [file genes-08-00270-s001.zip › Figure S11.jpg]

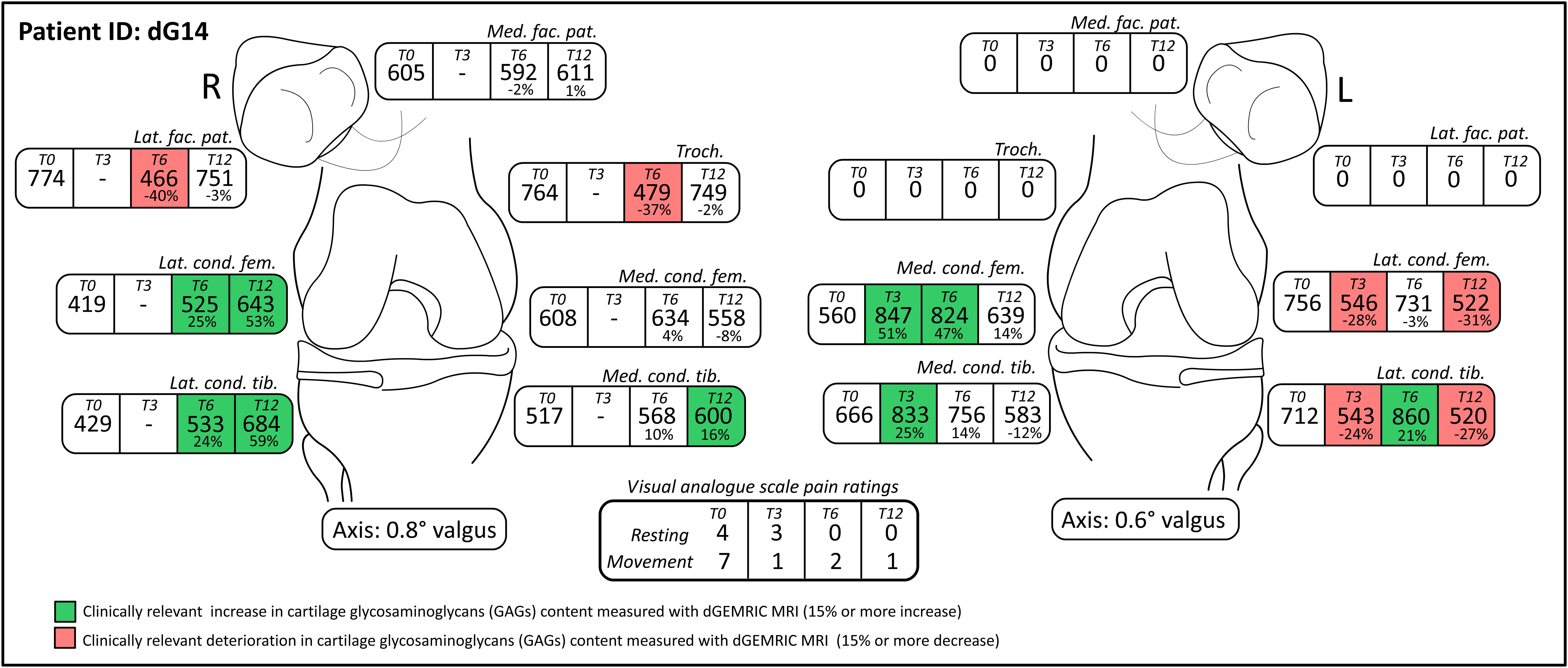

Supplement: Supplementary file 1 [file genes-08-00270-s001.zip › Figure S12.jpg]

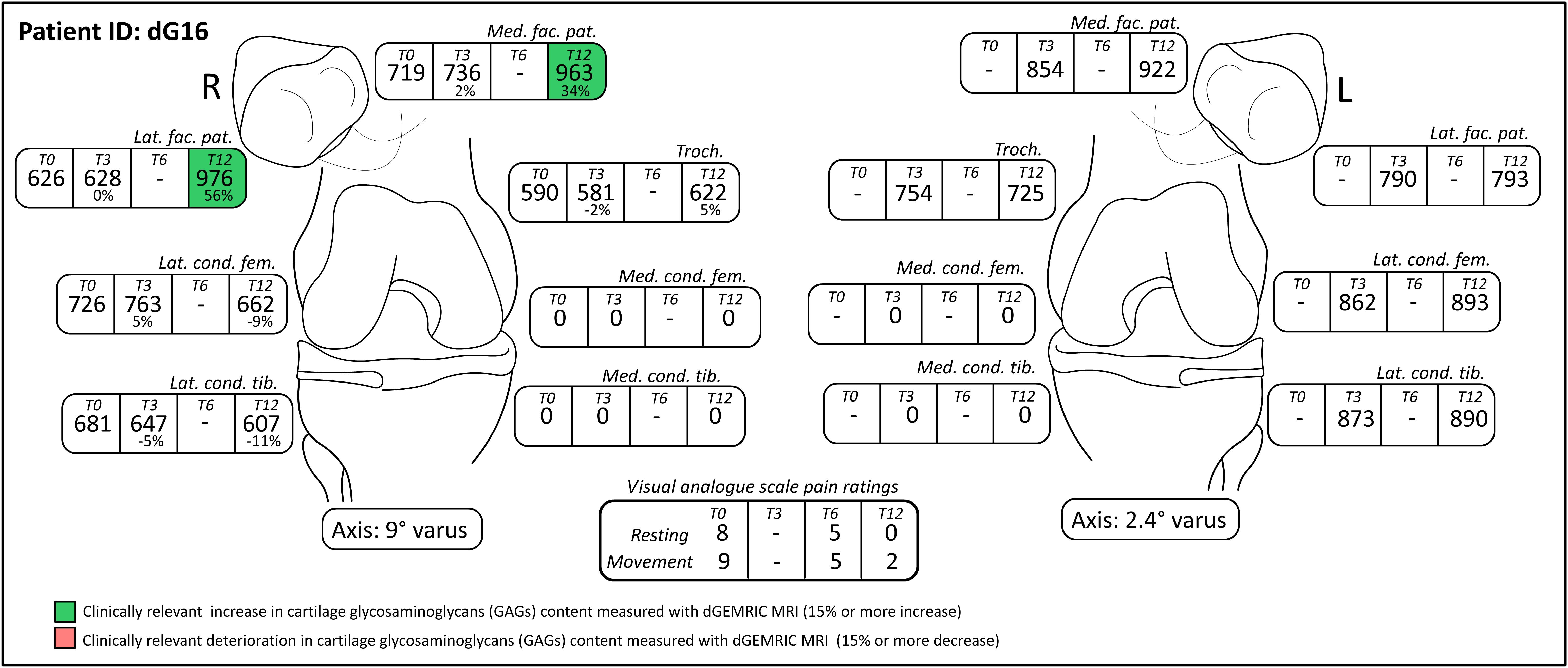

Supplement: Supplementary file 1 [file genes-08-00270-s001.zip › Figure S13.jpg]

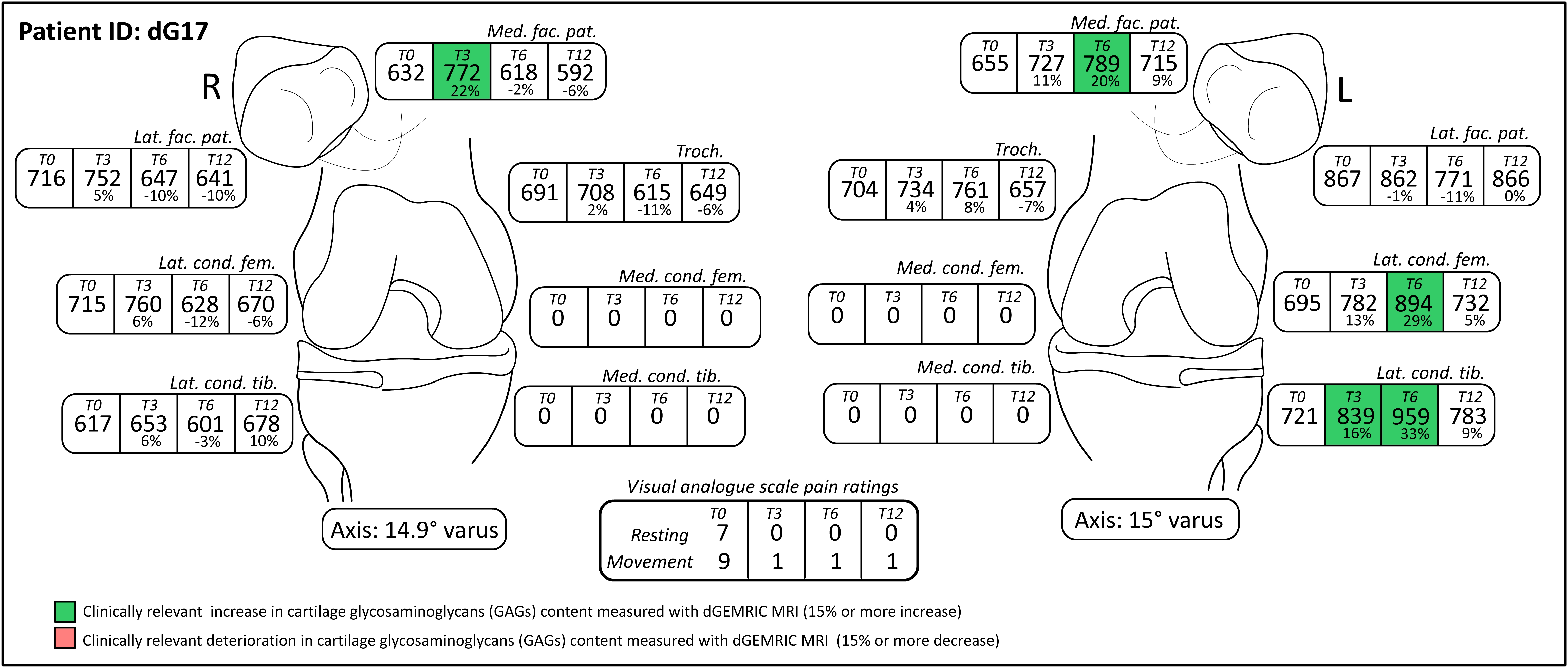

Supplement: Supplementary file 1 [file genes-08-00270-s001.zip › Figure S14.jpg]

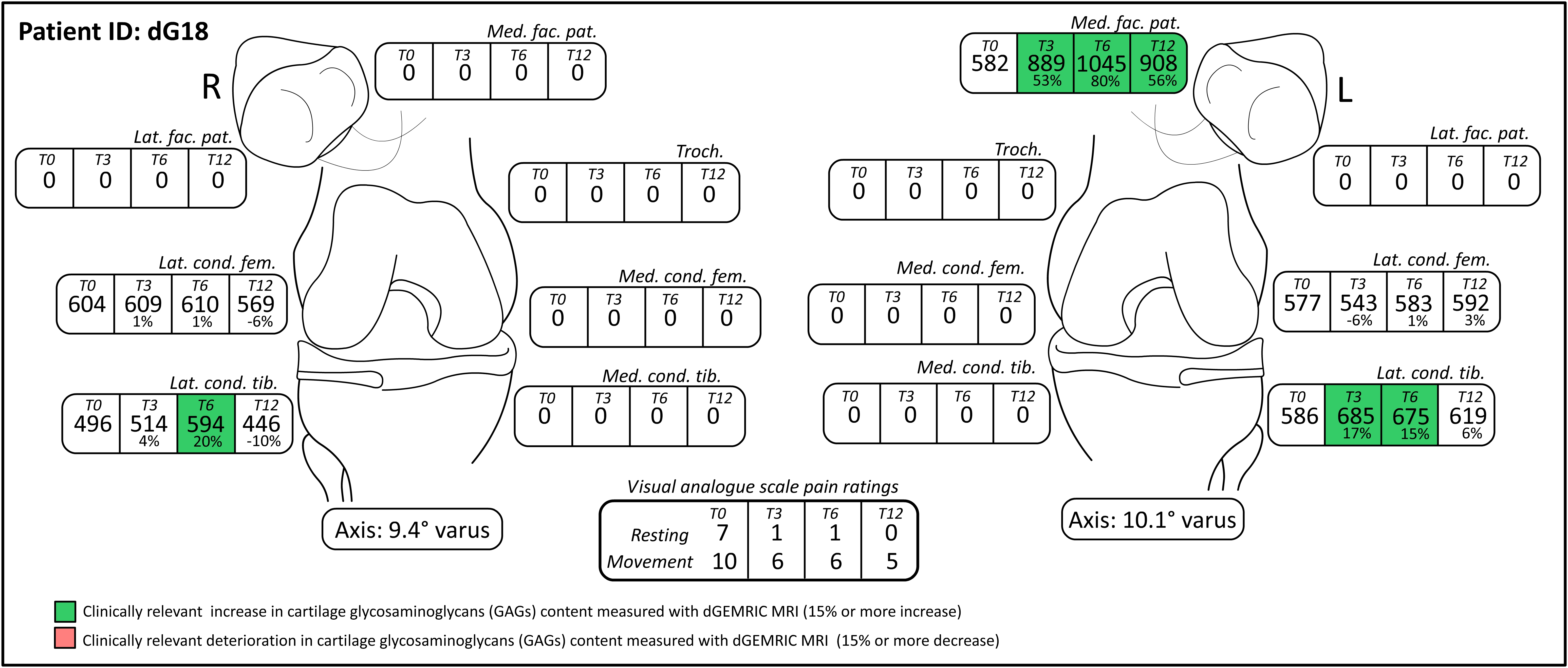

Supplement: Supplementary file 1 [file genes-08-00270-s001.zip › Figure S15.jpg]

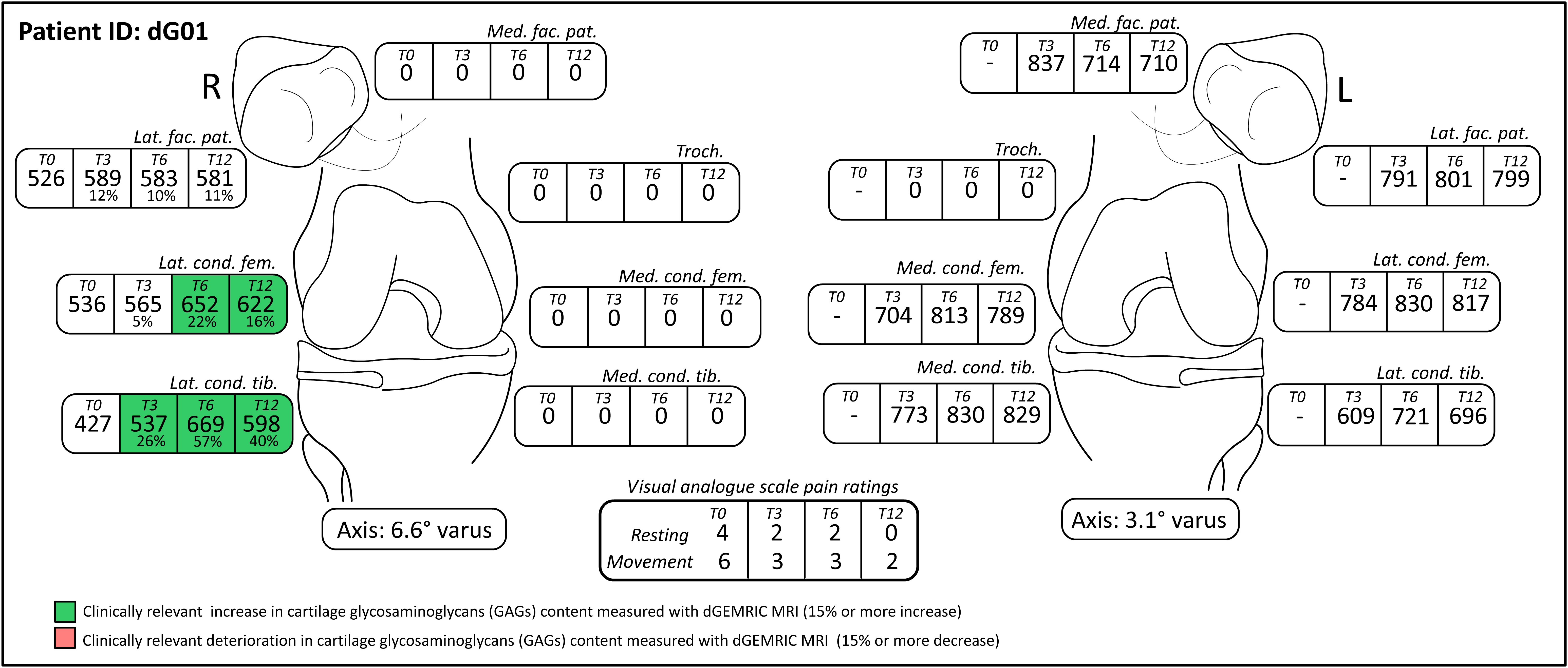

Supplement: Supplementary file 1 [file genes-08-00270-s001.zip › Figure S1.jpg]
